# Hyperspectral reflectance-based phenotyping for quantitative genetics in crops: Progress and challenges

**DOI:** 10.1016/j.xplc.2021.100209

**Published:** 2021-05-27

**Authors:** Marcin Grzybowski, Nuwan K. Wijewardane, Abbas Atefi, Yufeng Ge, James C. Schnable

**Affiliations:** 1 Center for Plant Science Innovation and Department of Agronomy and Horticulture, University of Nebraska-Lincoln, Lincoln, NE, USA; 2 Department of Plant Molecular Ecophysiology, Institute of Plant Experimental Biology and Biotechnology, Faculty of Biology, University of Warsaw, Warsaw, Poland; 3 Department of Biological Systems Engineering, University of Nebraska-Lincoln, Lincoln, NE, USA; 4 Department of Agricultural Biological Engineering, Mississippi State University, Starkville, MS, USA

**Keywords:** hyperspectral reflectance, phenotyping, quantitative genetics, maize

## Abstract

Many biochemical and physiological properties of plants that are of interest to breeders and geneticists have extremely low throughput and/or can only be measured destructively. This has limited the use of information on natural variation in nutrient and metabolite abundance, as well as photosynthetic capacity in quantitative genetic contexts where it is necessary to collect data from hundreds or thousands of plants. A number of recent studies have demonstrated the potential to estimate many of these traits from hyperspectral reflectance data, primarily in ecophysiological contexts. Here, we summarize recent advances in the use of hyperspectral reflectance data for plant phenotyping, and discuss both the potential benefits and remaining challenges to its application in plant genetics contexts. The performances of previously published models in estimating six traits from hyperspectral reflectance data in maize were evaluated on new sample datasets, and the resulting predicted trait values shown to be heritable (e.g., explained by genetic factors) were estimated. The adoption of hyperspectral reflectance-based phenotyping beyond its current uses may accelerate the study of genes controlling natural variation in biochemical and physiological traits.

## Teaser

This review discusses the potential applications of trait data derived from hyperspectral reflectance information to quantitative genetics. The heritability of traits predicted from hyperspectral reflectance data in genetically diverse backgrounds across years and treatments is demonstrated. Collecting hyperspectral reflectance data from large association panels across multiple environments has the potential to enable GWAS and studies of genotype by environment interactions *in silico*.

## Quantifying plant traits using hyperspectral reflectance data

When light strikes the surface of a plant, it will experience one of three fates. First, the light can be absorbed by the plant, by photosynthesis, converted to heat, or re-emitted as fluorescence (van Bezouw et al., 2019). Second, the light can be reflected by the plant, or third, it can be transmitted through the plant and emerge out the other side. The probability of each of these fates varies depending on the wavelength of the light and the properties of the plant the light is striking (Kumar et al., 2002). Hyperspectral imaging generally captures information about the intensity of light reflected from the plant across many specific wavelengths. To interpret this information it is important to know the relative intensity of the light striking the plant at different wavelengths. Different hyperspectral imaging technologies take different approaches when addressing this question, including providing their own light source with known properties, using standard panels with known reflective properties in images, or including a second sensor facing the opposite direction of the main sensor to directly measure the intensity of the incoming light at different wavelengths directly. The technical details of how hyperspectral measurements can be made are beyond the scope of this review, but have been well explained elsewhere (Bruning et al., 2020).

Two broad approaches can be taken for deploying measuring plant phenotypes from hyperspectral data. The first is to identify a small set of wavelengths where individual values or ratios are informative. These include, for example, normalized difference vegetation index (Rouse et al., 1974) or photochemical reflectance index (Gamon et al., 1992). These indices rely on spectral regions that are well-known absorption peaks maxima for important plant pigments: chlorophyll and carotenoids. Exposing plants to stress often leads to changes in concentration of these pigments and also to changes in vegetation index. This makes such indices robust tools when it comes to obtaining general information about plant status. The primary advantages of this approach are, first that sensors can be made at lower cost, and second that the interpretation of the resulting models is straightforward. The alternative approach is to employ sensors that measure many specific wavelengths, either including only the visible spectrum (350–700 nm) or expanding to include the near infrared (700–1100 nm), and sometimes shortwave infrared (1100–2500 nm). The cost of devices capable of collecting these data is higher but declining over time. The primary advantages are, firstly that predictions using values from the full spectrum improve accuracy, even for traits with well-known reflectance maxima, such as chlorophyll (Yendrek et al., 2017); and secondly that the same set of hyperspectral data can be analyzed to quantify multiple traits. In addition, hyperspectral data collected in previous years can be reanalyzed with newly trained models, mining additional information and insight from already conducted experiments.

**Figure 1:**
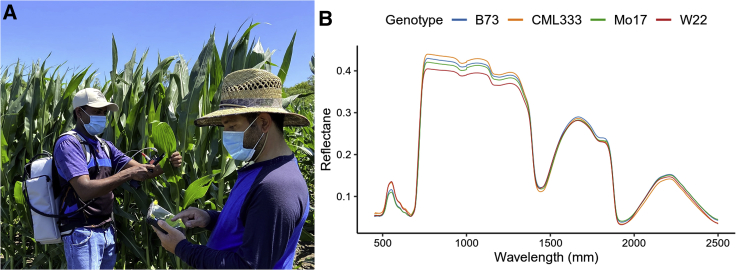
Collection of hyperspectral reflectance data in a maize genetics experiment. **(A)** Use of a portable and battery-powered spectroradiometer to collect hyperspectral reflectance data as part of a maize field experiment conducted in the summer of 2020. **(B)** Variation in patterns of hyperspectral reflectance observed among the leaves of four distinct maize inbred genotypes. Each datapoint consisted of the measurements of 2151 distinct spectral intensities between 350 and 2500 nm in wavelength.

Hyperspectral data can be collected from satellites, from unmanned aerial vehicles (UAVs) or planes, from cameras on the ground, or from handheld spectrometers in direct contact with the plant surface. There are several important differences between handheld spectrometers and hyperspectral camera carriers on different platforms. First, handheld spectrometers usually measure larger numbers of discrete wavelengths. Second, measurements are more accurate because those types of devices have artificial light sources and offer constant calibration. Third, handheld spectrometers conduct leaf-level (point) measurements while spectrometers on other platforms conduct canopy-level measurements. This is a crucial difference, since canopy structure itself can lead to changes in the hyperspectral profile (Knyazikhin et al., 2013). Effects of canopy structure can be minimized with vector normalization (Knyazikhin et al., 2013; Wang et al., 2020b) or using LiDAR (light detection and ranging) data (Ewald et al., 2018). However, no such type of research has been done solely on crop ecosystems (Wang et al., 2020b) and it will be required before spectrometers mounted on UAVs can be used routinely in genetics and agriculture. While collecting data with handheld spectrometers is much more time consuming compared with other platforms, previously published research demonstrates that it is possible to collect data from hundreds of individuals (Yendrek et al., 2017; Ge et al., 2019), making this technology suitable for the scale required in genetic studies. Moreover, by eliminating the confounding effects of variation in illumination and leaf angle relative to the camera, data collected by handheld spectrometers require much less pre-processing than imaging-based systems. Since handheld spectrometers currently have lower cost, technology, and skill barriers to entry we will concentrate on the use of this technology in this review (Figure 1).

A range of approaches to training models used to predict plant traits from hyperspectral data have been employed. Two of the most widely used at the moment are partial least squares regression (PLSR) (Wold et al., 2001) and least absolute shrinkage and selection operator regression (Tibshirani, 1996). These approaches have been able to predict a wide range of biochemical and physiological traits from hyperspectral data. In recent years, a growing number of studies have demonstrated the use of hyperspectral reflectance data from spectrometers to build models that can predict a range of plant traits (Table 1). These studies have been conducted in different species, although maize and wheat have been common targets, with the goal of predicting different output traits from the same initial data type. The number of paired ground truth and hyperspectral reflectance data points collected in individual studies varies dramatically, from 61 to 2478 samples. This variation reflects the varying degrees of difficulty required to collect ground truth measurements for different target traits. Common targets of prediction have included specific leaf area and its inverse leaf mass per unit area (Serbin et al., 2011, 2019; Silva-Perez et al., 2017; Yendrek et al., 2017; Ely et al., 2019; Ge et al., 2019) and nitrogen content (Serbin et al., 2011; Silva-Perez et al., 2017; Yendrek et al., 2017; Ely et al., 2019; Ge et al., 2019; Wang et al., 2020a). Furthermore, studies have also demonstrated the potential of training models to predict the abundance of different sugars and non-structural carbohydrates (Ely et al., 2019), phosphorous and other macro and micro nutrient abundance (Silva-Perez et al., 2017; Ge et al., 2019), the abundance of a wide range of metabolites (Vergara-Diaz et al., 2020), and even water use efficiency (Cotrozzi et al., 2020) from hyperspectral reflectance data. One of the factors that has attracted the most attention is the potential to estimate photosynthetic parameters, such as the maximum rate of carboxylation of ribulose bisphosphate (V_cmax_) (Serbin et al., 2011; Silva-Perez et al., 2017; Yendrek et al., 2017; Fu et al., 2019; Wu et al., 2019; Wang et al., 2020a). The conventional approach to collecting measurements of many photosynthetic parameters is to fit non-linear models to data obtained from gas-exchange measurements (Farquhar et al., 1980). However, these measurements require expensive equipment to collect and significant amounts of time per data point. Even with the most recent photosynthesis measurement devices, a single measurement requires at least 5 min and typically 20–30 min (Stinziano et al., 2019). This substantially constrains the study of genetic determinants of natural variation in photosynthetic parameters within species, as if significant genotype × environment interactions (G×E) exist for many of these parameters. One of the first publications to indicate the possibility of using hyperspectral reflectance data to estimate photosynthetic parameters more rapidly than was possible from conventional gas-exchange phenotyping was by Serbin et al. (2011). The authors demonstrated the ability to predict values of both V_cmax_ and J_max_—the maximum rate of ribulose bisphosphate regeneration—with R^2^ values of ~0.9 in a 78-sample dataset collected from 11 tree species across three temperature regimes. Variation between species is frequently lager, and therefore easier to predict, than variation between individuals of a single species. However, more recent work has demonstrated that it is also possible to predict V_cmax_ and J_max_ variation among individuals of a single species by employing hyperspectral reflectance data collected from several hundred individuals of maize (Yendrek et al., 2017) and wheat (Silva-Perez et al., 2017), although with somewhat lower R^2^ values than were obtained for between species predictions.

**Table 1:** Summary of 11 research papers which use hyperspectral reflectance to predict various traits.

Reference	Species	Phenotype	R^2^	Sample size	Modeling method
Serbin et al. (2011)	aspen and cotton wood tree	leaf mass per area	0.95	78	PLSR
		Nitrogen	0.89	78	
		maximum rates of RuBP carboxylation (V_cmax_)	0.89	78	
		maximum rates of RuBP regeneration (J_max_)	0.93	78	
Yendrek et al. (2017)	maize	Chlorophyll	0.85	268	PLSR
		Nitrogen	0.95	203	
		specific leaf area	0.67	182	
		maximum rates of RuBP carboxylation (V_cmax_)	0.65	214	
		Sucrose	0.6	61	
Heckmann et al. (2017)	maize	maximum rate of the A-Ci curve	0.69	50	PLSR
		carbon to nitrogen ratio	0.89	50	
		initial slope of the A-Ci curve	0.58	50	
	Brassica	aximum rate of the A-Ci curve	0.51	50	
		carbon to nitrogen ratio	0.90	50	
	Moricandia (mixed species)	maximum rate of the A-Ci curve	0.44	50	
		carbon to nitrogen ratio	0.80	50	
		initial slope of the A-Ci curve	0.65	50	
Silva-Perez et al. (2017)	wheat	Nitrogen	0.93	525	PLSR
		leaf mass per area	0.98	525	
		Chlorophyll	0.81	614	
		maximum rates of RuBP carboxylation (V_cmax_)	0.74	488	
		maximum rates of RuBP regeneration (J_max_)	0.70	488	
		nitrogen content per unit leaf area (N_mass_)	0.86	615	
		phosphorus content per unit leaf area	0.65	431	
		maximum rubisco activity normalized to 25°C (V_cmax25_)	0.62	488	
		Rate of CO_2_ assimilation	0.49	560	
		V_cmax25_/N_mass_	0.40	488	
		Phosphorus	0.40	431	
		stomatal conductance	0.50	560	
Serbin et al. (2019)	diverse species	leaf mass per area	0.89	2478	PLSR
Wu et al. (2019)	tropical tree	maximum rubisco activity normalized to 25°C (V_cmax25_)	0.89	216	PLSR
Ely et al. (2019)	eight eudicot species	Nitrogen	0.92	178	PLSR
		Carbon	0.95	178	
		carbon to nitrogen ratio	0.92	177	
		leaf mass per area	0.90	179	
		leaf water content	0.89	179	
		Protein	0.85	177	
		amino acids	0.58	174	
		Nitrate	0.51	179	
		Starch	0.80	174	
		total non-structural carbohydrates	0.70	177	
		total sugars	0.69	179	
		Sucrose	0.76	177	
		Glucose	0.56	177	
		Fructose	0.44	179	
Ge et al. (2019)	maize	Chlorophyll	0.94	846	PLSR or SVM
		leaf water	0.70	846	
		specific leaf area	0.55	846	
		Nitrogen	0.86	846	
		Phosphorus	0.44	846	
		Potassium	0.59	846	
Fu et al. (2019)	tobacco	maximum rates of RuBP carboxylation (V_cmax_)	0.75	212	Regression stacking
		maximum rates of RuBP regeneration (J_cmax_)	0.63	212	
Vergara-Diaz et al. (2020)	durum wheat	74 metabolites	0–0.81	360	LASSO
Cotrozzi et al. (2020)	maize	rate of CO_2_ assimilation	0.84	180	PLSR or LASSO
		Transpiration	0.83	180	
		stomatal conductance,	0.73	180	
		intercellular CO_2_ concentration	0.51	180	
		instantaneous water use efficiency	0.69	180	
		intrinsic water use efficiency	0.44	180	
		leaf temperature	0.89	180	
		Chlorophyll	0.61	180	
		leaf water potential	0.63	180	
		leaf osmotic potential	0.60	180	
		leaf osmotic potential at full turgor	0.53	180	
Wang et al. (2020a)	maize	Chlorophyll	0.95	178	PLSR
		Nitrogen	0.96	351	
		maximum rates of RuBP carboxylation (V_cmax_)	0.81	298	

R^2^ values are based on validation dataset. PLSR, partial least squares regression; LASSO, least absolute shrinkage and selection operator; SVM, support vector machine.

A number of studies have also demonstrated the ability to predict the abundance of a range of inorganic nutrients and plant metabolites from hyperspectral reflectance data. Ely et al. (2019) quantified the abundance of 9 different metabolites across roughly 180 samples drawn from 8 plant species. Ely et al. were able to successfully construct models to predict the abundance of starch and sucrose with relatively high accuracy (R^2^ > 0.75), while the accuracy was lower for glucose and fructose (R^2^ < 0.60). The same study observed that the total protein content can be predicted with high accuracy (R^2^ > 0.8). As with photosynthetic parameters, a key question was whether the prediction accuracy of models based on data from multiple species could be replicated with data from a single species. In a study employing data from a maize association panel grown in three environments, Ge et al. (2019) demonstrated the ability to predict leaf nitrogen, phosphorus, and potassium content with good accuracy from hyperspectral reflectance data. Using 360 samples from durum wheat, Vergara-Diaz et al. (2020) demonstrated the ability to predict the gas chromatography-mass spectrometry-measured abundance of at least 15 metabolites with acceptable performance (R^2^ > 0.5) in each of three tissues—leaves, lemmas, and glumes—using models trained on hyperspectral reflectance data. Those metabolites have played roles in physiological functions, such as photosynthesis metabolism, carbon partitioning, and storage (sucrose and glucose); osmotic adjustment and stress tolerance (raffinose, maltose, glycerol, and proline); photorespiration intermediates (glycerine and serine); and organic acids related to osmoprotection and respiratory metabolism (malate and fumarate). Various metabolites in this study have shown poor predictive performance, such as lysine, glycine, and fucose. There are several possible explanations: they do not produce any differentiable or appreciable spectral absorption, their signals are masked by signals from other traits (such as chlorophyll or water), or their content or inter-sample variation was too small to be accurately quantified. Since only four genotypes were employed in this study, the latter reasons seem to be likely, and research on larger sample sizes are needed to exclude this possibility. Similar to the earlier multi-species study (Ely et al., 2019), prediction accuracy for fructose was again inferior to that of sucrose. This suggests that the properties that make a trait feasible to predict, or not as the case may be, may be generalizable across species and studies. Several studies have demonstrated the ability to accurately estimate leaf water content from hyperspectral reflectance data (Ely et al., 2019; Ge et al., 2019). However, hyperspectral reflectance data may be able to predict more features related to plant water use than simply instantaneous water content. A study employing six maize hybrids with diverse degrees of drought stress tolerance demonstrated that traits, including stomatal conductance and leaf temperature, in addition to relative water content, could be predicted from hyperspectral reflectance data (R^2^ > 0.7). In the same study, leaf water potential and osmotic potential could be predicted with accuracies in the range of R^2^ from 0.5 to 0.7 (Cotrozzi et al., 2020). As a dataset of only 180 paired hyperspectral and ground truth datapoints were employed, it may be possible to increase the prediction accuracy for these water use-related traits by employing models trained with larger datasets.

Taken together, the above examples demonstrate that hyperspectral data are able to effectively estimate values for a wide range of plant traits of interest to plant geneticists and plant breeders. However, as shown above, efforts to develop and validate these approaches have been driven primarily by biochemical and physiological applications. The application of hyperspectral data to address quantitative genetics challenges, such as mapping genes while controlling within-species variation for traits of interest, will first require evaluation of whether or not predicted values are heritable, that is if the variation in them can be explained by genetic factors.

## Challenges in quantitative genetics

Once it is possible to accurately measure a trait across hundreds of individuals of a target species, quantitative genetic tools can be used to identify regions of the genome or specific genes controlling variation in the target trait. Similarly, traits scored across hundreds of individuals can be used to train genomic prediction models that can guide the breeding of new varieties with improved values for the target trait. Traits which are expensive to measure or that require significant labor per data point are less likely to be targets of quantitative genetic investigations even when those same traits may be valued by farmers, consumers, or policy makers. Plant nutrient status, photosynthetic capacity, and stress tolerance are all traits which are of value to agriculture, but investigations of genetic regulators and breeding of these characteristics have been slowed by the expense of collecting data (nutrient status), the time-consuming nature of data collection (photosynthesis), or the logistical challenges of creating repeated and equivalent environmental stresses. Adding to the challenge, plant nutrient status, photosynthesis, and metabolism are all sensitive to environmental perturbations. As a result, quantitative genetic analysis of genes controlling these traits requires the collection of data from hundreds of individuals not once, but repeatedly across many diverse environments.

Studies of the genetic control of easily measured plant traits can demonstrate the size and complexity of data collection necessary to employ quantitative genetic tools, such as the genome-wide association study (GWAS) in plant species. In addition to the effort required to accurately measure a trait, another key metric which determines the effectiveness of GWAS is the heritability of the trait in question (Miao et al., 2019). Heritability is an estimate of the proportion of total variance for a given trait in a population that is explained by genetic variation between individuals in that population. Two types of heritability can be calculated: narrow sense and broad sense. Narrow-sense heritability reflects only additive effects. For sets of unrelated genotypes, narrow-sense heritability can be calculated from genotype means or unreplicated data from individuals. However, calculating narrow-sense heritability requires the availability of genetic marker information. Broad-sense heritability incorporates additive, dominance, and epistatic genetic effects. In contrast to narrow-sense heritability, broad-sense heritability can be calculated for unrelated sets of genotypes in the absence of genetic marker information, provided that individual genotypes are replicated. In principle, broad-sense heritability is a superset of narrow-sense heritability and should always be an equal or higher number. However, the estimation of heritability is imprecise and influenced by experimental, technical, and quantitative factors (Lynch and Walsh, 1998), so this will not always be the case.

The ideal trait for breeding or gene mapping is characterized by high heritability. For example, the broad-sense heritability of both flowering time and plant height of maize in a nested association mapping (NAM) population are estimated to be >0.9 (Peiffer et al., 2014). However, successful GWAS have also been conducted in maize for metabolites related to carbon and nitrogen metabolism with low to moderate heritability (0.14–0.68) (Zhang et al., 2015). It should be noted that the NAM population used in this study was very large (*n* > 4000). Using a smaller population of 289 diverse individuals, Riedelsheimer et al. (2012) were only able to map the quantitative trait locus (QTL) for biochemical compounds that have repeatabilities higher than 0.63. This indicates that mapping genes for a trait with lower heritability requires either greater replication, larger mapping populations, or a more favorable genetic architecture (Miao et al., 2019). Heritability values are not fixed and they can change during developmental stages (Liang et al., 2017; Miao et al., 2020) or because of time of exposure to stress (Chen et al., 2014; Feldman et al., 2018). This is also true for QTL effect and it is known that a given QTL can have different impacts on a given trait at different stages in development (Muraya et al., 2017; Feldman et al., 2018). Yet, as a result, the high cost and labor-intensive nature of phenotypic data collection, GWAS using trait data from the same population at multiple time points remains the exception rather than the rule.

Since the concept of association mapping was introduced to plant biology (Thornsberry et al., 2001), it became one of the most important tools to link genomic regions with various phenotypes. Lots of attention was given to decoding the genetic architecture of different morphological and developmental traits, such as flowering time in maize (Buckler et al., 2009), various agronomic traits in rice (Huang et al., 2010), height of maize (Peiffer et al., 2014) and sorghum (Miao et al., 2020), or the root architecture of maize and sorghum (Zheng et al., 2020). Because these types of traits are relatively easy and cheap to measure, it is not uncommon that they are evaluated in many environments and in a few different populations. Such data provide clear benefits. Using data from three big maize populations (Ames, Chinese, and US-NAM), (Li et al., 2016) conducted a large-scale GWAS on maize flowering time data from multiple environments. For days to anthesis they were able to find 77 QTL, among which only 18 overlapped between CN and US-NAM, whereas for days to silk they found 78 QTL, with 19 overlapped, respectively. This results clearly demonstrate that, using a single population for QTL mapping, may be not sufficient to obtain the full picture of the genetic architecture of the trait of interest. However, generating such amounts of physiological and biochemical data is much more expensive and technically challenging.

Alongside morpho-developmental traits, various studies were done on biochemical and physiological traits. Zhang et al. (2015) analyzed 12 key carbon and nitrogen metabolites in a US-NAM population using 100 000 enzymatic assays. They were able to identify 514 candidate genes, among which extensive pleiotropy were found. However, this research was done based on samples from one year and one location, so the level of environmental effect and G×E remains unknown. The same population was used for analyzing 20 elements in kernel composition from plants grown in four different environments (Ziegler et al., 2017). Variance partitioning reveals massive G×E effect for every element. In each case, more than half of the observed phenotypic variation was explained by the G×E effect. These contrast with traits, such as height or flowering time. A study on the same maize NAM population found that, for these traits, more than half of the variation was explain by genotype effect and only 0.2 by G×E in the case of flowering time and about 0.1 in height (Peiffer et al., 2014). The importance of environmental effects on traits related to elements also came from the work of Yang et al. (2018). They measured 17 elements across a diverse panel of 529 rice accessions in two locations in three parts of plants and found 72 loci responsible for controlling variation in these traits. While 30 QTL were common across environments, 42 were specific for one place. Results from this work clearly show that measurements across multiple environments have to be done to fully understand the genetic architecture underlining composition of elements in various plant tissues.

Perhaps the most difficult traits to quantify are those related to abiotic and biotic stress response. Stress in general affects many traits simultaneously; however, effect size on various traits can be different. It is known that many studies tend to use very high levels of stress in experiment design and concentrate on traits that are largely affected by the given stressor (Claeys et al., 2014). However, less attention has been given to mild stress, which is much more common in nature. Moreover, it is not uncommon for different stresses to appear in various parallel combinations. When a plant is exposed to such conditions during its whole life cycle no obvious response may appear; however, the impact on yield may be substantial. Therefore, proper tools to study mild stress should be improved.

Another difficulty in measuring stress response is time dependency. Depending on how long a plant was exposed to the stressor, the effects on phenotypes might be different. So far, only a small number of studies measure GWAS on plant stress response traits during different time points (Campbell et al., 2015; Guo et al., 2018; Ubbens et al., 2020). These types of studies are technically challenging, especially in terms of biochemical traits, since those measurements are usually destructive. This makes them difficult to monitor over time without substantially increasing the sample size. Hyperspectral reflectance offers the great opportunity to predict many of the discussed traits in a nondestructive manner, allowing researchers to monitor stress response over time. Moreover, because many traits can be predicted from single measurements, this offers opportunities to study plant responses to stress at biochemical and physiological levels. However, to be useful in genetic research, this prediction has to be heritable. To our very best knowledge, this very important problem has not yet been addressed in literature.

## Traits estimated from hyperspectral data are heritable

As shown above, many researchers have demonstrated the ability to predict trait values for a range of various biochemical and physiological plant properties using hyperspectral reflectance data. However, these studies primarily focus on the overall accuracy of prediction and cross-validation within a single dataset. With the exception of Wang et al. (2020a), current studies generally do not test out of sample datasets and do not estimate the heritability of the predicted trait values produced by analysis of hyperspectral reflectance data. To address both of these questions we employed published data from Ge et al. (2019) collected from greenhouse- and field-grown plants of the Buckler-Goodman maize association panel (Flint-Garcia et al., 2005) in 2018, and a second set of previously unpublished data collected using the same protocol from field-grown plants in 2019. The Buckler-Goodman panel has been resequenced, providing high-density SNP marker data that enable the calculation of narrow-sense heritability (Bukowski et al., 2017). A detailed description of methods used in this analysis can be found in the supplemental information.

**Figure 2:**
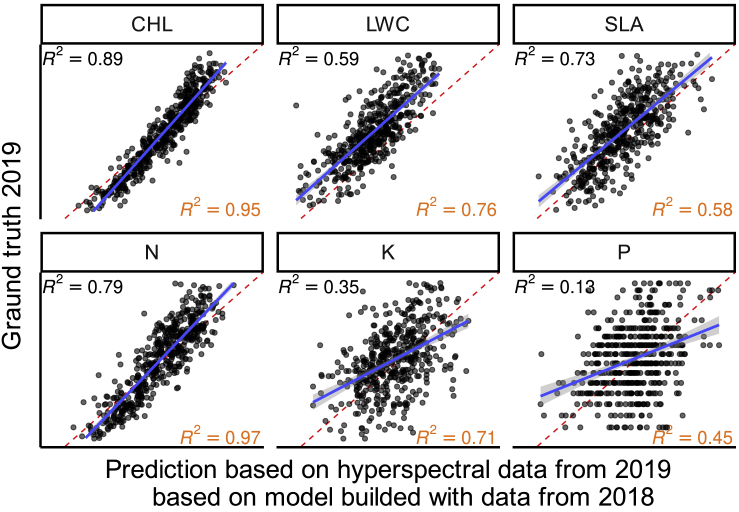
Evaluation of model performance built from data from 2018 (Ge et al., 2019) on data from 2019. Upper left R^2^ values show coefficient of determination for presented data, whereas bottom right R^2^ values are obtained by cross-validation on 2018 data (Ge et al., 2019). CHL, chlorophyll content; LWC, leaf water content; SLA, specific leaf area; N, nitrogen content; K, potassium content; P, phosphorus content.

In both 2018 and 2019 data from field-grown plants were collected from high- and low-nitrogen application field plots. We focused on ground truth data for six different traits: abundance of chlorophyll, nitrogen, phosphorus, potassium in leaf tissue, leaf water content, and specific leaf area (mass per unit area). We built prediction models using 2018 data and evaluated the performance of these models using new reflectance and ground truth data collected in 2019. Good correlations between predicted and ground truth data were observed for chlorophyll and nitrogen content, moderate correlation for specific leaf area and leaf water content and rather low correlation for potassium and phosphorous (Figure 2). Of the six traits evaluated, chlorophyll, specific leaf area, and nitrogen all exhibited only modest declines in prediction accuracy relative to estimates of prediction accuracy obtained from cross-validation within the 2018 dataset (Ge et al., 2019). The accuracy with which models trained on 2018 leaf water content data could predict 2019 leaf water content was substantially lower than estimates from cross-validation within the 2018 data (R^2^ = 0.59 versus 0.76). Prediction accuracy for the abundance of potassium and phosphorous showed the greatest declines in between years versus within year prediction accuracy (R^2^ = 0.35 versus 0.71 and R^2^ = 0.13 versus 0.45). While potassium, phosphorous, and leaf water content exhibited greater declines between years prediction accuracy, it may still prove possible to train models that generalize well across environments employing expanded sets of training data collected across a wider range of environments. A good example of the potential of such an approach is the work of Serbin et al. (2019), who were able to achieve a prediction accuracy for specific leaf area of R^2^ = 0.89 by incorporating data collected from multiple species using different spectrometer instruments across 11 environments. A recent study also found that published ecological models developed for a range of species in eastern North America (Wang et al., 2020b) were able to predict within-species variation in chlorophyll and nitrogen in maize with accuracies of R^2^ = 0.88 and R^2^ = 0.85, respectively (Wang et al., 2020a). However, studies of the transferability of models remain rare and more work is needed to fully evaluate the potential unified and transferable models to predict the same traits in different environments and species. However, in the cases of chlorophyll, specific leaf area, and nitrogen, existing prediction models for maize are already providing consistent accuracy across years and environments (Figure 2). The question that naturally arises is how heritable are these estimates of plant leaf properties? How much of the observed variance is explained by genetic factors?

**Figure 3:**
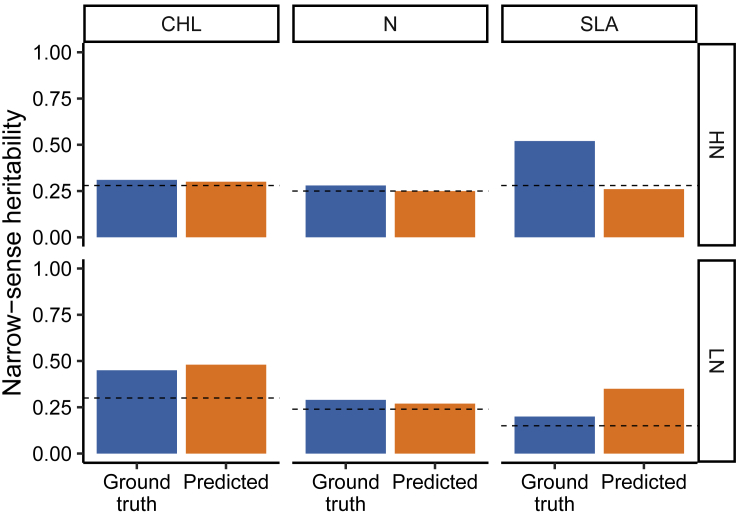
Comparison between narrow-sense heritability for ground truth and predicted from spectra values. Dashed lines indicate expected narrow-sense heritability value obtain by multiplying ground truth narrow-sense heritability value with R^2^ values from model performance evaluation. CHL, chlorophyll content; N, nitrogen content; SLA, specific leaf area; HN, high nitrogen condition; LN, low nitrogen condition.

Correlations between predicted and ground truth values across an entire dataset collected in multiple environments do not necessarily indicate that predicted measurements will be under strong genetic control. The proportion of variance explained by genotype to genotype variation will vary among traits even when considering ground truth data. For traits where environmental differences—in this case high- and low-nitrogen treatments—explain a large proportion of total variance, it would be possible for a model to achieve significant predictive value only by learning to distinguish between plants grown under different treatment conditions, while not learning how to predict between plant variation in a single environment. Narrow-sense heritability in high- and low-nitrogen environments was first calculated from ground truth measurements collected in 2019. If ground truth measurements are perfectly accurate and disagreement between ground truth and predicted values are explained solely by random error, the maximum narrow-sense heritability of estimated trait values derived from hyperspectral reflectance data should be equal to the product of narrow-sense heritability and the R^2^ observed between ground truth and predicted trait values (Figure 3). In five of six cases evaluated, the heritability of trait values estimated from hyperspectral reflectance data equalled or exceeded the product of these two values. This suggests one or both of two conclusions. Firstly, ground truth measurements are unlikely to be perfectly accurate. In fact, it is quite plausible for models trained on large amounts of noisy data to exceed the prediction accuracy of the data used to train them. Secondly, errors in phenotyping data are unlikely to be entirely random. For example, more than half the error in maize biomass estimates from image data is explained by genotype to genotype variation in the size and direction of error (Liang et al., 2017). In general, for some traits, current models for predicting trait values using hyperspectral reflectance data appear to be as good, or nearly as good, as methods used for ground truth data collection, at a fraction of the cost and time of conventional methods. The narrow-sense heritability of trait estimates derived from hyperspectral reflectance data is sufficiently high that it should indeed be possible to identify genes controlling trait variation within single environments and, with properly replicated studies, genes controlling genotype × environment variation across multiple environments or treatments.

## Future perspectives

The adoption of trait value estimation from hyperspectral data has the potential to significantly benefit both quantitative genetics and plant breeding. A significant barrier to adoption is a communication gap between those who are developing and testing predictive models, and those who could potentially employ the same models in quantitative genetic and plant breeding contexts. Here, we summarize several approaches to accelerate the adoption and deployment of hyperspectral reflectance phenotyping in plant quantitative genetics contexts. We urge researchers developing new models to employ experimental designs that make it possible to calculate broad-sense heritability from repeatedly measuring genetically identical individuals. Broad-sense heritability tends to provide a more reliable and stable estimate of the genetic contribution to variance than estimates of narrow-sense heritability derived from marker data of unrelated populations of individuals. Reporting estimates of heritability, regardless of broad sense or narrow sense, in concert with the correlation between ground truth and predicted values would benefit plant science researchers substantially in evaluating which models for which traits are worth incorporating into their research or breeding programs. Obviously genetic study is not the direct goal of many researchers. However, the calculation and reporting of heritability values, regardless of broad sense or narrow sense, in concert with the correlation between ground truth and predicted values would substantially benefit those researchers interested in genetic investigations. It may also be a relatively low effort to increase the reuse and citation of studies that are already being conducted.

A second question of substantial interest to both quantitative geneticists and breeders is how well models trained using existing data will perform in new years or new locations. There is no easy answer. More data is always better, but, unlike incorporating the calculation of heritability, the decision to collect more data points across more environments entails a substantial increase in the total time and resources required to complete a given study. One partial solution would be to encourage the implementation of open science conventions widely adopted in genomics and metabolomics for the deposition and sharing of raw datasets. Conventions on data deposition and sharing in phenomics are much less well defined because of the much greater diversity of data types that fall within the broad umbrella of phenomics data. Phenomics is a label that can apply to anything from a simple flat text with numerical gas-exchange measurements, to archives of tens of thousands of RGB images, LiDAR point clouds, or hyperspectral data cubes (Yang et al., 2020). By comparison, hyperspectral reflectance data collected from spectrometers is one of the easier data types to share and disseminate. The structure of the data lends itself well to being shared in flat text files with ground truth measurements incorporated as part of the same data frame. Files incorporating records from even thousands of individuals are still sufficiently small to be deposited in open data repositories, such as Zenodo, Figshare, or DataDryad, or in dedicated repositories for hyperspectral data, such as the Ecological Spectral Information System (ecosis.org) (Wagner et al., 2018). While this repository is primarily targeted at ecological studies, at least one research group succeeded in using models originally constructed in an ecological research context to predict within-species variation in maize (Wang et al., 2020a). The accumulation of publicly available hyperspectral data would not only aid in evaluating model performance across years and locations, but also accelerate the training of robust multi-species, multi-environment models for various physiological and biochemical traits similar to those demonstrated for specific leaf area (Serbin et al., 2019).

Currently, the majority of models trained to predict phenotypes from hyperspectral data employ PLSR (Serbin et al., 2011; Silva-Perez et al., 2017; Yendrek et al., 2017; Ely et al., 2019; Cotrozzi et al., 2020). This approach works well in a wide range of cases. However, there may be room to further improve prediction accuracy through the evaluation of additional machine learning algorithms. For example, Fu et al. (2019) found that a support vector machine showed the best prediction for maximum rate of carboxylation of ribulose bisphosphate (V_cmax_) in tobacco (R^2^ = 0.67), while PLSR has the lowest prediction performance among six compared methods with R^2^ = 0.60. Moreover, regression stacking, a technique that is used to mix different predictors to improve prediction accuracy (Wolpert, 1992), improves the R^2^ to 0.75. Software packages, such as caretEnsemble in R, can automate the process of fitting various models and exploring parameter space, reducing the additional work required to test and evaluation of a range of models (Deane-Mayer and Knowles, 2019). The potential of future algorithmic innovations to train more accurate models from the same datasets is another motivation to ensure the effective annotation and storage of hyperspectral reflectance data and associated ground truth measurements.

One challenge to the wider adoption of hyperspectral phenotyping for genetics and breeding is that the models to predict traits from these data are often essentially "black boxes" without a clear understanding of the underlying mechanisms at play. In some cases it is possible to understand the workings of the model by estimating variable importance across different hyperspectral wavelengths (Wold et al., 2001). Those values indicate how important a given wavelength is in predicting the value for a particular trait. This approach works well for pigments, which have known absorption light maxima and interpretation is straightforward. However, for traits which we do not have such a well-defined interactions with light, interpreting results might be much more difficult. While the black-box nature of trait prediction based on hyperspectral data is not inherently a problem for the use of hyperspectral phenotyping in genetics research, genetics may be able to help address this issue. Studies on rice demonstrated that it is possible to successfully identify casual loci for a particular wavelength or spectral index (Feng et al., 2017; Sun et al., 2019). When casual loci are identified and the function of the underlying gene is known, this knowledge can help interpret the biological source of variation in a given wavelength and thus provide insight on the biological role of this wavelength in prediction of a given trait (see Box 1).

Box 1 **Potential aid of hyperspectral phenotyping for quantitative genetics and vice versa**

How hyperspectral phenotyping can aid quantitative genetics:

• Quantitative genetics requires a lot of measurements
• Many agriculturally or biologically important traits are expensive or slow to score
• Many research groups cannot execute large multi-environment field trials
• Hyperspectral reflectance data collected from large association populations grown in multiple environments can enable *in silico* GWAS

How quantitative genetics can aid hyperspectral phenotyping:

• The mechanism underling hyperspectral prediction for many traits is unclear
• The lack of clear and established mechanisms slows adoption
• GWAS conducted using hyperspectral phenotypes can identify specific genes with known functions that may shed light on the mechanistic basis for hyperspectral phenotyping of specific traits

The genetic mechanisms responsible for controlling many biochemical and physiological traits in plants remain partially or completely unknown. Quantitative genetic studies leveraging natural genetic variation have enabled the identification of genes controlling a wide range of plant properties. Hyperspectral reflectance data have the potential to substantially expand the range of traits studied using quantitative genetics, including many biochemical and physiological traits where the genes controlling natural variation remain partially or completely unknown. However, it would be a mistake to think of this as simply a new technology for measuring individual phenotypes. The real long-term potential of this technology is that the same reflectance data can be employed by different models to estimate a wide range of related or unrelated traits. Hyperspectral reflectance data collected from a large replicated GWAS population by one research group to study the genes responsible for variation in nitrogen uptake efficiency could, with a properly trained model, be employed by another research group to map genes controlling variation in water use efficiency, and by a third interested in the genes regulating the accumulation of various specialized metabolites. It is this potential for reusable phenotypic datasets to address distinct research questions, just as early QTL mapping and association populations created the potential for reusable genotypic datasets, that makes the potential of hyperspectral reflectance phenotyping to both expand our current genetic knowledge and address the challenges of breeding for the 21st century so exciting.

## Data availability

Spectral reflectance data and ground truth measurements have been deposited in https://doi.org/10.21232/y5TTxY3N.

## Funding

This research was supported by the Office of Science (BER), 10.13039/100000015U.S. Department of Energy, grant no. DE-SC0020355 to J.C.S. and Y.G., the 10.13039/100000001National Science Foundation under grant OIA-1557417 to Y.G. and J.C.S. and OIA-1826781 to J.C.S. This project was completed utilizing the Holland Computing Center of the University of Nebraska, which receives support from the Nebraska Research Initiative.

## Author contributions

M.G., Y.G., and J.C.S. conceived of the study. N.K.W., A.A., and Y.G. conducted the experiments and collected the data. M.G. analyzed the data. M.G. and J.C.S. wrote the manuscript. All authors read and approved the final manuscript.

## Supplemental information

Document S1. Supplemental methods and supplemental references

Document S2. Article plus supplemental information
