# Supplementary material for: Hyperspectral reflectance-based phenotyping for quantitative genetics in crops: Progress and challenges
Source: Plant Commun. 2021 May 27;2(4):100209. doi: 10.1016/j.xplc.2021.100209 (PMC8299078; doi:10.1016/j.xplc.2021.100209)
Supplement: Document S1. Supplemental methods and supplemental references [file mmc1.pdf]

Plant Communications, Volume *2*

Supplemental information

Hyperspectral reflectance-based phenotyping for quantitative genetics in crops: Progress and challenges

Marcin Grzybowski, Nuwan K. Wijewardane, Abbas Atefi, Yufeng Ge, and James C. Schnable

Material and methods

Data Source

Ground truth and spectra data for the 2018 experiment were sourced from Ge et al. (2019). Ground truth and spectral data for the 2019 experiment were collected and processed following the same methodology described in Ge et al. (2019).

In brief, the Buckler-Goodman maize 282 association panel (Flint-Garcia et al., 2005) were planted at Havelock Research Farm of the University of Nebraska-Lincoln (40.853 N, 96.61 W) on June 1st 2019. The diversity panel was replicated in two conditions, one under low nitrogen condition (− N) and the other under normal condition (+ N). For the + N treatment, 135 kg/ha urea (dry fertilizer) was applied; whereas for the − N treatment, no supplemental N fertilizer was applied. Leaf sampling was conducted when 50% of plants were flowering. Second or third representative leaves (counting down from top) were cut, placed in Ziploc bags, and stored in a cooler and transported to the lab for analysis.

VIS–NIR–SWIR reflectance spectra of leaf samples were measured using a benchtop spectroradiometer (FieldSpec4, Malvern Panalytical Ltd.). Three measurements per leaf were collected with range 350–2500 nm and a spectral sampling interval of 1 nm. For further analysis spectra collected from the same leaf were averaged.

Chlorophyll measurements were conducted using a handheld chlorophyll concentration meter (MC-100, Apogee Instruments, Inc., Logan, UT) with three measurements per leaf.

Leaf area was measured with a leaf area meter (LI-3100, LI-COR Biosciences, Lincoln, NE). Fresh weight (FW) of the leaves was measured using a digital balance. After initial data collection, leaf samples were placed in a walk-in oven set to 50 °C and dried over 72 h after which the dry weight (DW) values of each leaf were recorded.. Leaf Water Content (LWC, %) was calculated as (FW–DW)/FW × 100%. Specific Leaf Area (SLA, m^2/kg) was calculated as LA/DW.

Dried samples were sent to a commercial lab (Midwest Laboratories, Inc., Omaha, NE) where nitrogen (N), phosphorus (P) and potassium (K) content were determined.

Both, ground truth and spectra data used in this analysis were deposited in:
https://doi.org/10.21232/y5TTxY3N

Tratis modeling

All analyses were performed in R (R Core Team, 2020). Similar to the approach described in Ge et al. (2019), spectral values below 450 nm were removed and a Savitzky–Golay smooth filter were applied to the data to reduce noise (window size equal to 5 and polynomial size 2; Savitzky and Golay, 1964). Next, individual 1 nm measurements were averaged into bins of five measurements (5 nm), reducing the total number of potential predictive variables.

Partial Least Squares Regression (PLSR; Wold and Eriksson, 2001) was employed to build predictive models for all traits based on data from 2018. Modeling was performed using the pls (Mevik et al., 2020) and caret packages (Kuhn, 2020) in R. Prior to the modeling, each predictor variable was zero centered and scaled to unit variance. One hundred sets of five-fold cross validation was used to choose the optimal number of latent variables as well as to avoid model over-fitting. For latent variables, values from one to 30 were tested and the model with the smallest value of cross-validated root mean squared error in the 2018 dataset was applied to previously unseen 2019 data. Finally, ground truth data from 2019 were regressed on predicted values to obtain the coefficient of determination (R^2) for each phenotype.

Heritability estimation

Genotype data for the maize association panel were obtained from www.panzea.org (Bukowski et al., 2018). Data for inbred lines presented in this study were subset from the VCF files and filtered with following criteria: minor allele frequency > 5% and missing value < 5% using bcftools (Danecek et al., 2021). Next, VCF files were converted to bed format and variants in strong local linkage disequilibrium were pruned using Plink 1.9 (Chang et al., 2015). Pruning was done with sliding window size 500 bp, step 500 bp and cutoff was set to r2 > 0.2. Next, kinship was computed on the remaining marker set in GEMMA (Zhou and Stephens, 2012).
For each trait, a mixed linear model was fit with kinship as a random factor using the mmer function from sommer package (Covarrubias-Pazaran, 2016). Narrow-sense heritability was defined as the proportion of variance explained by kinship (genetic variance) relative to the total variance, according to the equation:

$$h^2 = \sigma^2_g / (\sigma^2_g + \sigma^2_e)$$

Where σ^2_g is genetic variance and σ^2_e is residual variance.

Bibliography:

Ge, Y., Atefi, A., Zhang, H. *et al.* High-throughput analysis of leaf physiological and chemical traits with VIS–NIR–SWIR spectroscopy: a case study with a maize diversity panel. *Plant Methods* 15, 66 (2019)

Flint-Garcia SA, Thuillet AC, Yu J, Pressoir G, Romero SM, Mitchell SE, Doebley J, Kresovich S, Goodman MM, Buckler ES. Maize association population: a high-resolution platform for quantitative trait locus dissection. Plant J. 2005;44(6):1054–64.

Savitzky A, Golay MJE. Smoothing and differentiation of data by simplified least squares procedures. Anal Chem. 1964;36:1627–39.

R Core Team. R: a language and environment for statistical computing. Vienna: R Foundation for Statistical Computing; 2020. https://www.R-project.org/.

Wold S, Sjöström, M, Eriksson, L. PLS-regression: a basic tool of chemometrics. *Chemometrics and Intelligent Laboratory Systems*. 2001; 58(2): 109–130.

Bjørn-Helge M, Ron W, Kristian HL. pls: Partial Least Squares and Principal Component Regression. R package version 2.7-3. https://CRAN.R-project.org/package=pls

Kuhn M. caret: Classification and Regression Training. R package version 6.0-86. https://CRAN.R-project.org/package=caret

Bukowski R, Guo X, Lu L, Zou C, He B, Rong Z, Wang B, Xu D, Yang B, Xie C, Fan L, Gao S, Xu X, Zhang G, Li Y, Jiao Y, Doebley JF, Ross-Ibarra J, Lorant A, Buffalo V, Romay MC, Buckler ES, Ware D, Lai J, Sun Q, Xu Y. Construction of the third-generation *Zea mays* haplotype map, *GigaScience*, 2018; (7)4:gix134

Danecek P, Bonfield JK, Liddle J, Marshall J, Ohan V, Pollard MO, Whitwham A, Keane T, McCarthy SA, Davies RM, Li H, Twelve years of SAMtools and BCFtools, *GigaScience*, 2021; 10(2):giab008,

Chang CC, Chow CC, Tellier LCAM, Vattikuti S, Purcell SM, Lee JJ (2015) Second-generation PLINK: rising to the challenge of larger and richer datasets. GigaScience, 2015; 4

Zhou X, Stephens M. Genome-wide efficient mixed-model analysis for association studies. *Nature Genetics,* 2014 44:821–824.

Covarrubias-Pazaran G. Genome assisted prediction of quantitative traits using the R package sommer. PLoS ONE, 2016; 11(6):1-15.
